# Quantifying Center-level Aggressiveness in Transplanting Suboptimal, Deceased Donor Kidneys in the United States

**DOI:** 10.1097/TXD.0000000000001886

**Published:** 2026-01-12

**Authors:** Teresa Po-Yu Chiang, Mackenzie A. Eagleson, Jennifer D. Motter, Michelle R. Krach, Hannah C. Sung, Nicholas L. Wood, Dorry L. Segev, Darren E. Stewart, Allan B. Massie, Jacqueline M. Garonzik-Wang

**Affiliations:** 1 Department of Surgery, NYU Grossman School of Medicine, New York City, NY.; 2 Department of Surgery, Johns Hopkins University School of Medicine, Baltimore, MD.; 3 Department of Epidemiology, Johns Hopkins School of Public Health, Baltimore, MD.; 4 Department of Obstetrics, Gynecology and Women's Health, Saint Louis University School of Medicine, St. Louis, MO.; 5 Chronic Disease Research Group, Hennepin Healthcare Research Institute, Minneapolis, MN.; 6 Department of Medicine, Hennepin Healthcare, University of Minnesota, Minneapolis, MN; 7 Department of Surgery, University of Wisconsin School of Medicine and Public Health, Madison, WI.

## Abstract

**Background.:**

Understanding center-level decision-making for suboptimal kidney (SOK) offers is critical to ensure utilization of all transplantable kidneys.

**Methods.:**

We quantified center-level variation in accepting SOK deceased donor kidney transplant (DDKT) offers using 2021–2023 national registry data. SOK subtypes included: donor age >60, ultimate cold ischemia time >24 h, hepatitis C positive, terminal serum creatinine >2.0 mg/dL, donation after circulatory death, kidney donor profile index >85%, and public health service increased risk donors. Gini coefficient (Gini) was used to analyze inequality in DDKT utilization by SOK subtype. Multilevel logistic regression models were used to calculate the median odds ratio (mOR), measuring center-level variation in accepting SOK donor offers among adult centers.

**Results.:**

Of all DDKTs, 72.6% were from donors with at least 1 SOK characteristic. Inequality persisted in utilization of SOK DDKTs (Gini of all SOKs: 0.53, Gini of all non-SOKs: 0.47). The 193 adult centers accepted a median (interquartile range) of 12.5% (8.4%–19.2%) offered non-SOK donors and 7.2% (4.6%–10.8%) offered SOK donors. Non-SOK donors and SOK donors were refused by a median (interquartile range) of 5 (3–10) and 9 (4–23) centers, respectively. The SOK subtypes with the least and the most center-level variance in acceptance were increased risk donor (mOR = 2.06) and cold ischemia time >36 h (mOR = 4.86), respectively.

**Conclusions.:**

Centers vary sharply in their willingness to accept certain types of SOK offers. Informing centers of their patterns of accepting specific donor phenotypes compared with their peers may motivate centers to accept more SOKs for clinically suitable recipients, thus improving patient access to DDKT.

Only a quarter of US deceased donor kidney transplant (DDKT) waitlisted candidates receive a DDKT within 5 y, even with the rising number of donors and after implementation of kidney allocation system (KAS).^1^ Providers are often faced with the choice of whether to wait for a better kidney or to accept a suboptimal kidney (SOK) for any given candidate. Despite the need, a national study using data from 2008 to 2015 by Husain et al^2^ found that 84% of all accepted kidneys were refused at least once by centers before placement, and 92.6% of the refusals were attributed to donor or organ quality concerns. The presence of Systems Improvement Agreements might have further complicated offer acceptance behavior,^3^ although the recent removal of survival rate metrics from the Centers for Medicaid and Medicare’s conditions of participation might help mitigate risk aversion.^4^

Historically, there has been considerable variation in utilization of SOKs,^5-11^ likely secondary to variation in offer acceptance practices.^2,3,7,10-14^ However, most evidence on this topic was collected before KAS, a 2014 allocation policy change which substantially altered allocation priority in the United States.^15-20^ Moreover, in recent years, there has been a growing body of evidence demonstrating favorable outcomes for many types of SOKs,^6,21-53^ including kidneys from donors >70 y old,^48^ from donors who had hepatitis C virus (HCV),^41,42,45^ and from donors with acute kidney injury (AKI).^6,33-39,47^ Specifically, studies have reported a survival benefit associated with accepting kidneys from even donors >80 y old,^23^ donation after circulatory death (DCD),^26,32^ donors with kidney donor profile index (KDPI) >85%,^27,29,45,53^ public health service increased risk donors (IRDs),^40^ and donors who had HCV.^43,44^ Furthermore, the Organ Procurement and Transplantation Network (OPTN) launched a new kidney allocation policy on March 15, 2021, prioritizing candidates listed within 250 nautical mile radius from the donor hospital. Therefore, prior studies on offer acceptance may not generalize to the present, as some centers might have adapted their acceptance practices accordingly over time. Moreover, prior work measured overall aggressiveness by aggregating acceptance patterns across an array of donor characteristics, essentially viewing donor quality as a single characteristic and dichotomizing centers as either aggressive or nonaggressive.^5^ Yet, “aggressiveness” is not necessarily uniform across these dimensions nor binary. For example, a center may aggressively accept kidneys from older donors, but not those who had HCV. Understanding and quantifying center-level variation in acceptance of specific kinds of SOKs in the modern era is therefore necessary to design center-specific strategies for encouraging increased utilization of SOKs.

The Scientific Registry of Transplant Recipients (SRTR) compares each center’s offer acceptance practices to the national average in their Program-Specific Reports. The reports provide case-mix adjusted acceptance ratios for all kidney offers as well as certain subgroups, including offers from low-, medium-, or high-KDRI donors, and offers whose sequence was >100, indicating how likely a center was to accept those offers in the previous year compared with the national average. However, there is limited information on center-level offer acceptance patterns for other specific potentially transplantable subtypes of SOKs.

We sought to quantify US center-level differences in SOK acceptance after the policy change in March 2021 using 2021–2023 national transplant registry data. We hypothesized that inequality in the utilization of SOKs persists and that substantial variations in center aggressiveness exist but not uniform across different SOK subtypes. We also hypothesized that there would be center-level correlations in aggressiveness between different SOK subtypes. Our goal was to inform efforts aimed at encouraging centers to accept more SOK offers, considering the potential survival benefits for medically suitable waitlisted candidates.

## MATERIALS AND METHODS

### Data Source

This study used data from the SRTR. The SRTR data system includes data on all donor, waitlisted candidates, and transplant recipients in the United States, submitted by the members of the OPTN. The Health Resources and Services Administration, U.S. Department of Health and Human Services provides oversight to the activities of the OPTN and SRTR contractors. This dataset has previously been described elsewhere.^54^ This study was classified as exempt and received approval by the NYU Grossman School of Medicine Institutional Review Board.

### Deceased Donor Population

We studied all deceased kidney donors for whom at least 1 kidney was transplanted to an adult recipient between April 1, 2021, and December 31, 2023. Dual, en bloc, and multiple organ transplants were excluded. Donors with a positive serology of anti-HIV antibody or RNA nucleic acid test were excluded. We defined “suboptimal kidney (SOK)” as a kidney from a deceased donor who met any of the following characteristics: age 60 y or above; DCD; terminal serum creatinine (SCr) >2.0 mg/dL; positive test for HCV (HCV+; positive anti-HCV antibody or HCV RNA); designated as an IRD (based on 2013–2020 Centers for Disease Control and Prevention criteria)^55^; KDPI score >85%; or prolonged, laterality-specific cold ischemia time (CIT) exceeding 24 h. Kidneys from deceased donors who did not meet any SOK criteria were defined as non-SOKs.

We used 2, complementary approaches to quantify center-level inequity in using or accepting various types of SOKs, the first examining center-level variation in transplanting SOK DDKTs, the second estimating center-level variation in rates of accepting SOK offers from donors whose kidneys were eventually transplanted. The definition of SOKs was the same in both analyses, except for CIT, because of the lack of laterality-specific information at the time of each offer. Therefore, for the offer acceptance analysis, only donors whose transplanted kidneys all had CIT >24 h were deemed to have had prolonged CIT.

### Center-level Variation Among SOK Transplants

To quantify center-level inequality in utilization of SOKs for unilateral DDKTs, we calculated the Gini coefficient using various cutoff criteria for donor age (>60, >65, >70), DCD and age (>50, >55, >65, >70), KDPI (>85, >90, >95), terminal SCr (>2.0, >2.5, >3.0, >3.5, >4.0, >4.5, >5.0 mg/dL), and CIT values (>24, >30, >36) as SOK categories. Additional SOK subtypes in combination were analyzed to evaluate donors with multiple suboptimal characteristics: SCr >2.0 and age >50 y old, DCD and CIT >24 h, and KDPI >85% and CIT >24 h. The Gini coefficient measures how the distribution of performed DDKT departed from perfect equality among all centers, with the value of 0 indicating perfect equality (all SOK DDKT were evenly distributed among all centers) and 1 indicating perfect inequality (a single center performed all the DDKTs in an SOK category).^8^ For comparison, we calculated the overall Gini coefficient including all DDKTs, and the Gini coefficient of exclusively non-SOKs, as baseline measures that reflect the disproportionality in overall center volumes. The purpose was to compare the Gini coefficient of SOK categories to the Gini coefficient of the non-SOKs, to see if the distribution of SOK DDKTs was more unequal than the distribution of non-SOK DDKTs. We used both the jackknife method and bootstrapping (with 1000 iterations) to evaluate whether the difference between the Gini coefficient of SOK versus non-SOK reached statistical significance. Lorenz curves were created to visually examine disproportionality in center-level kidney utilization. Centers with greater volume contributed more to the Gini coefficient and to a bigger uptick in the Lorenz curve. Cutoff points such as donor age >75 y, or DCD donor age >70 y, were explored but not included in final analyses because of the rarity of the SOK category within the study period.

### Acceptance of SOK Offers

For each of the above-mentioned DDKT donors for whom at least 1 kidney was eventually transplanted, we evaluated how centers responded to their kidney offers, stratified by SOK categories as defined in “Deceased donor population.” We analyzed the “Y” (acceptance) and all the “N” (refusal) responses from centers. Although centers refusing for a candidate below the final acceptor in the match run sequence did not have a chance to accept a kidney for that patient, we considered the refusal as evidence of disinterest in the donor. Provisional acceptances (“Z”) and bypassed candidates (“B”) were excluded, but if a center accepted an organ out of sequence, it was treated as any other acceptance. On the donor level, we excluded from the analyses donors who had multiple records of offer acceptances in different match runs (83/7581 donors in 2021, 85/10 673 donors in 2022, 46/11 130 in 2023 offer data) because of the idiosyncrasies often surrounding the acceptance or refusal of these donor offers. On the center level, we excluded 28 pediatric centers (defined as centers for which >50% of candidates were age <18 at the time of listing) and 9 small centers whose annual DDKT volume was <1 annually during the study period. On the offer level, we excluded the refusal of HCV+ kidney offers made to candidates who indicated unwillingness to accept HCV+ kidneys (n = 20 491/1 304 853 offers from donors who were HCV+). To capture the final center-level acceptance of SOK offers, we defined acceptance as whether a center accepted 1 or both kidneys from a given SOK donor. In other words, if a center had refused offers on behalf of multiple candidates but accepted at least 1 offer for 1 candidate, the center was credited to have accepted this SOK donor. Contrarily, centers who refused 1 or more offers from a donor without any acceptance were deemed to have refused the SOK donor. This approach resulted in 1 aggregate response (either acceptance or refusal) per center for each SOK donor included in the analysis, among centers accepting or refusing at least 1 offer from the donor.

### Center-level Variation in SOK Acceptance Among Offered Donors

To model center-level variation in acceptance of SOK donors, we used a separate multilevel logistic model for each donor category (SOK categories, non-SOK, or among all), adjusting for donor age, sex, ethnicity (binary, Hispanic, or Latino versus others), race (White, Asian, Black, multiracial, or others), body mass index (kg/m^2^), DCD, death because of cerebrovascular events or stroke (yes/no), history of hypertension, diabetes, or positive HCV RNA, without adjusting for the SOK donor category being modeled. For instance, the multilevel logistic model used to quantify center-level variation in accepting DCD SOK donors would not adjust for “DCD” in the model and that the multilevel logistic model used to quantify center-level variation in accepting SOK donors with KDPI >85% would only adjust for ethnicity in the model. For each multilevel model, centers were considered random effects, and we calculated the median odds ratio (mOR) and center-level empirical Bayes means to characterize center-level variation in acceptance, as previously described.^14,56,57^ The mOR represents the difference in odds of offer acceptance between 2 randomly selected centers. Pairwise Spearman correlation coefficients (ρ: Spearmen ρ) of the center-level odds ratios in accepting SOK donors were presented to illustrate patterns in center-level offer acceptance across different SOK subtypes.

### Statistical Analysis

All analyses were 2-sided, with a 2-sided α of 0.05. All analyses were performed using Stata 17.0/MP for Linux (College Station, TX).

## RESULTS

### DDKT With SOK Donor

There were 49 048 single kidney DDKTs during the study period, performed at 230 transplant centers. DDKTs using SOKs of any category constituted 72.6% of all DDKTs performed (n = 35 625/49 048; Table 1). The most prevalent SOK characteristic was DCD (34.7% of all DDKT), followed by CIT >24 h (26.1%), IRD (19.5%), SCr >2.0 (15.5%), HCV (10.5%), age >60 y (7.6%), and KDPI >85% (7.2%; Table 2).

**TABLE 1.** Deceased kidney transplant donor characteristics included in analysis of inequality (Gini coefficient), stratified by suboptimal kidney status

Donor characteristics	Non-SOK DDKTs performed	SOK DDKTs performed
n	13 423	35 625
Age, y, median (IQR)	39 (27–49)	42 (32–53)
Female, n (%)	5384 (40.1)	12 286 (34.5)
Race, n (%)		
Asian	434 (3.2)	758 (2.1)
Black	2246 (16.7)	4625 (13.0)
White	10 482 (78.1)	29 650 (83.2)
Multiracial, native, or Pacific	251 (1.9)	579 (1.6)
Unreported	10 (0.1)	13 (<0.1)
Hispanic, n (%)	2607 (19.4)	4820 (13.5)
BMI, median (IQR)	27.4 (23.4–32.2, n = 13 207)	27.8 (23.9–32.6, n = 34 856)
KDPI from SRTR, median (IQR)	33 (15, 53)	50 (29, 71)
HTN, n (%)	6874 (51.3)	17 910 (51.1)
DM, n (%)	7046 (52.5)	14 262 (40.4)
CIT, h, median (IQR)	16.9 (13–20.2)	21.3 (16.9–26)
SCr, mg/dL, median (IQR)	0.89 (0.67–1.2)	0.95 (0.67–1.72)
Died of CVA, n (%)	3283 (24.5)	6710 (18.8)

BMI, body mass index; CIT, cold ischemia time; CVA, cerebrovascular events or stroke; DDKT, deceased donor kidney transplant; DM, diabetes mellitus; HTN, hypertension; IQR, interquartile range; KDPI, kidney donor profile index; SCr, terminal serum creatinine; SOKs, suboptimal kidneys; SRTR, Scientific Registry of Transplant Recipients.

**TABLE 2.** Number of transplants in each suboptimal kidney category for inequality (Gini coefficient) analysis

Donor category, n	N (%) of DDKT performed	N (%) of centers used	Gini coefficient
Ref: non-SOK	13 423 (27.4)	228 (99.1)	0.47
Any SOK	35 625 (72.6)	214 (93.0)	0.53
Ref: Age ≤ 60 (y)	45 316 (92.4)	230 (100)	0.50
Age > 60	3732 (7.6)	184 (80.0)	0.61
Age > 65	1011 (2.1)	143 (67.7)	0.68
Ref: CIT ≤ 24 (h)^a^	36 270 (74.0)	229 (99.6)	0.48
CIT > 24	12 778 (26.1)	204 (88.7)	0.65
CIT > 30	4352 (8.9)	163 (70.9)	0.78
CIT > 36	1668 (3.4)	125 (54.3)	0.84
Ref: without HCV	43 880 (89.5)	230 (100)	0.49
Anti-HCV (+)	5129 (10.5)	153 (66.5)	0.69
HCV RNA (+)	2778 (5.7)	130 (56.5)	0.74
Anti-HCV or HCV RNA (+)	5168 (10.5)	153 (66.5)	0.69
Ref: SCr ≤ 2.0 (mg/dL)	41 443 (84.5)	230 (100)	0.49
SCr > 2.0	7605 (15.5)	191 (83.0)	0.66
SCr > 2.0 and age > 50	1240 (2.5)	133 (57.8)	0.72
SCr > 2.5	5549 (11.3)	173 (75.2)	0.69
SCr > 3.0	4216 (8.6)	163 (70.9)	0.72
SCr > 3.5	3283 (6.7)	154 (67.0)	0.74
SCr > 4.0	2582 (5.3)	146 (63.5)	0.75
SCr > 4.5	2066 (4.2)	140 (60.9)	0.76
SCr > 5.0	1592 (3.2)	123 (53.5)	0.78
Ref: not DCD	32 004 (65.3)	230 (100)	0.50
DCD	17 044 (34.7)	198 (86.1)	0.52
DCD and CIT > 24	4901 (10.0)	184 (66.4)	0.66
DCD and age > 50 (y)	5505 (11.2)	189 (82.2)	0.58
DCD and age > 55	3290 (6.7)	179 (77.8)	0.61
DCD and age > 60	1285 (2.6)	145 (63.0)	0.67
Ref: KDPI ≤ 85 (%)	45 496 (92.8)	230 (100)	0.50
KDPI > 85	3548 (7.2)	173 (75.2)	0.64
KDPI > 85 and CIT > 24	1021 (2.1)	125 (54.3)	0.76
KDPI > 90	1881 (3.8)	155 (67.4)	0.67
KDPI > 95	628 (1.3)	117 (50.9)	0.73
Ref: non-IRD	35 625 (72.6)	214 (93.0)	0.49
IRD	9585 (19.5)	207 (90.0)	0.55

^*a*^Laterality specific.

CIT, cold ischemia time; DCD, donated after circulatory death; DDKT, deceased donor kidney transplant; HCV, hepatitis C virus; IRDs, increased risk donors; KDPI, kidney donor profile index; Ref, reference; SCr, terminal serum creatinine; SOK, suboptimal kidneys.

### Center Utilization of SOK

**FIGURE 1.**
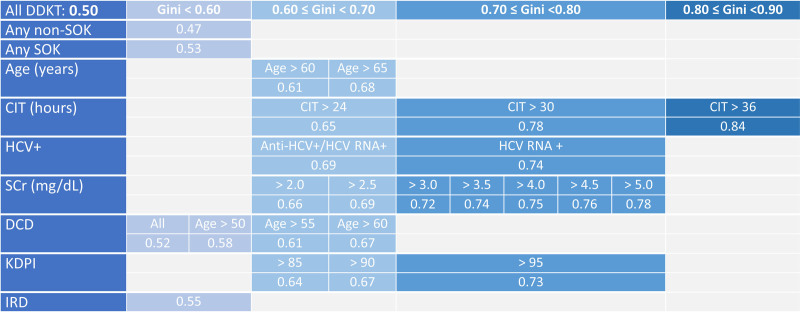
Heatmap highlighting the magnitude of inequality in the distribution of different SOK subtypes as demonstrated by Gini coefficient. Cells colored in darker blue reflect higher Gini values, whereas lighter blue cells have lower Gini values. CIT, cold ischemia time; DCD, donated after circulatory death; DDKT, deceased donor kidney transplant; Gini, Gini coefficient; HCV, hepatitis C virus; IRDs, increased risk donors; KDPI, kidney donor profile index; SCr, serum creatinine; SOKs, suboptimal kidneys.

**FIGURE 2.**
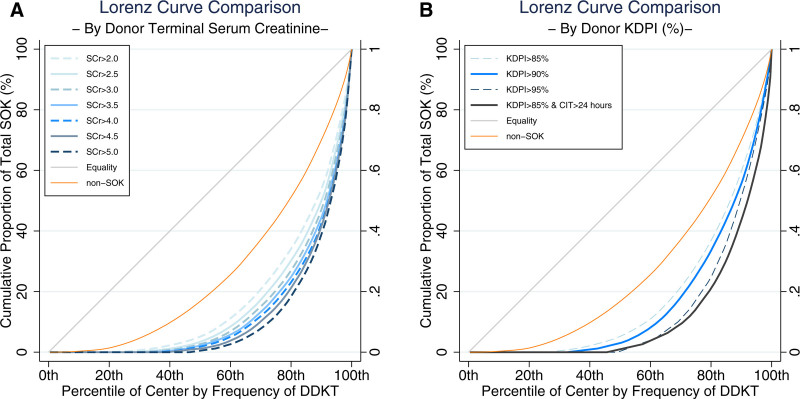
Graphical illustration of intercenter inequality by different cutoff criteria used in preoperative creatinine and KDPI. A, Donor terminal SCr cutoffs. B, Donor KDPI cutoffs. CIT, cold ischemia time; DDKT, deceased donor kidney transplant; KDPI, kidney donor profile index; SCr, serum creatinine; SOKs, suboptimal kidneys.

There were substantial inequalities in utilization of SOK DDKTs across all SOK categories (Table 2; illustrated graphically in Figure 1). While 90% (207/230) centers used kidneys from IRDs for DDKT, only 75.2% (173/230) centers used kidneys from KDPI >85% donors (Table 2). The overall Gini coefficient for single kidney transplants was 0.50, reflecting the overall center-level variation in transplant volume. SOK-specific values >0.50 indicate inequality in the distribution among centers in performing DDKTs of the specific SOK category, above and beyond the inequality in overall center volume. For instance, there was inequality in the distribution of DDKTs performed from donors with terminal SCr >2.0 mg/dL since the Gini coefficient was 0.66, which was numerically higher than 0.50. The inequality was more profound with more stringent age, CIT, SCr (Figure 2A), and KDPI cutoff levels (Table 2; Figure 2B). Nevertheless, the Gini coefficient of all SOKs (0.53) was greater than the Gini coefficient of all non-SOKs (0.47), and the difference (–0.06; 95% confidence interval, –0.09 to –0.02) was statistically significant (*P* = 0.001). This means that there was still inequality in performing DDKTs of any SOK category from 2021 to 2023, above and beyond what could have been explained as differences in overall center volume for performing non-SOKs (Table 3). The SOK categories with the highest Gini coefficients included CIT >36 h (0.84), positive HCV RNA (0.74), KDPI >95% (0.73), and those with elevated SCr >3.0 (0.69+).

**TABLE 3.** Median odds ratio and Gini coefficient for each suboptimal kidney category

Donor type	Donors studied (% of total donor)	% of total offers studied	Number of refused offers per donor, median (IQR)	Number of centers who refused per donor, median (IQR)	Total centers offered	Gini coefficient	Adjusted mOR
Total donors	28 498	(29 399 052)	59 (10–613)	8 (4–17)	193	0.50	2.02
Total non-SOK donors	9031 (31.7)	14.8	16 (6–94)	5 (3–10)	193	0.47	1.96
Total SOK donors	19 467 (68.3)	85.2	122 (16–1014)	9 (4–23)	193	0.53	2.13
Age > 60 (y)	2323 (8.2)	15.7	753 (116–2600)	22 (8–58)	193	0.61	2.49
Age > 65	671 (2.4)	4.8	1182 (220–3060)	28 (10–62)	188	0.68	2.65
CIT > 24 (h)	4262 (15.0)	35.2	595 (48–2842)	16 (6–54)	193	0.65	2.87
CIT > 30	1259 (4.4)	14.8	1313 (185–4918)	27 (9–72)	193	0.78	3.91
CIT > 36	423 (1.5)	5.8	1732 (314–5759)	40 (11–82)	193	0.84	4.86
Anti-HCV (+)	2933 (10.3)	4.3	55 (9–350)	7 (3–16)	170	0.69	2.40
HCV RNA (+)	1599 (5.6)	2.0	52 (10–303)	6 (3–14)	161	0.74	2.44
Anti-HCV (+) or HCV RNA (+)	2955 (10.4)	4.4	55 (9–350)	7 (3–16)	170	0.69	2.40
SCr > 2.0 (mg/dL)	4310 (15.1)	25.0	425 (63–1725)	13 (6–31)	193	0.66	2.86
SCr > 2.0 and age > 50	768 (2.7)	6.9	1322 (317–3577)	28 (11–62)	192	0.72	2.98
SCr > 2.5	3137 (11.0)	19.0	503 (80–1839)	14 (7–32)	193	0.69	3.24
SCr > 3.0	2372 (8.3)	14.8	565 (90–1946)	14 (7–33)	193	0.72	3.32
SCr > 3.5	1853 (6.5)	11.7	599 (110–1999)	15 (7–34)	193	0.74	3.24
SCr > 4.0	1464 (5.1)	9.4	649 (129–2076)	15 (7–35)	192	0.75	3.12
SCr > 4.5	1173 (4.1)	7.6	650 (145–2102)	15 (7–34)	192	0.76	3.17
SCr > 5.0	904 (3.2)	5.8	654 (148–2079)	16 (7–35)	191	0.78	3.40
DCD	9512 (33.4)	44.2	136 (19–1074)	10 (4–23)	191	0.52	2.17
DCD and CIT > 24	1523 (5.3)	16.0	1071 (170–4209)	24 (9–68)	191	0.66	2.93
DCD and age > 50 (y)	3213 (11.3)	21.7	503 (79–2179)	16 (8–45)	191	0.58	2.42
DCD and age > 55	1971 (6.9)	14.1	658 (109–2494)	20 (8–54)	191	0.61	2.59
DCD and age > 60	800 (2.8)	5.7	978 (163–2758)	22 (9–62)	190	0.67	2.78
KDPI > 85	2253 (7.9)	12.5	765 (151–2239)	24 (9–61)	192	0.64	2.52
KDPI > 85 and CIT > 24	443 (1.6)	3.5	1526 (553–3234)	48 (19–73)	191	0.76	3.58
KDPI > 90	1224 (4.3)	6.8	902 (178–2246)	27 (10–61)	190	0.67	2.60
KDPI > 95	435 (1.5)	2.7	1061 (294–2397)	34 (13–62)	190	0.73	2.47
IRD	5499 (19.3)	13.6	42 (9–353)	7 (3–14)	193	0.55	2.06

CIT, cold ischemia time; DCD, donated after circulatory death; Gini, Gini coefficient; HCV, hepatitis C virus; IQR, interquartile range; IRDs, increased risk donors; KDPI, kidney donor profile index; mOR, median odds ratio; SCr, terminal serum creatinine; SOKs, suboptimal kidneys.

### Characteristics of Offered SOK Donors

Of all 230 centers who performed DDKTs from 2021 to 2023, 193 predominantly adult-serving who received offers from 28 498 donors (Table 4; **Table S1**, **SDC**, https://links.lww.com/TXD/A810) were analyzed to quantify center aggressiveness in accepting SOK donor offers. For 66.3% of offers, the candidate’s refusal match run sequence number was greater than the sequence number of the final acceptor. Offers from donors with CIT >24 h constituted 35.2% of the total offers studied, although only 4262 of 28 498 (15.0%) of all donors studied had CIT >24 h (Table 3). On the other hand, offers from donors that tested positive for HCV constituted 4.4% of the offers, although 2955 of 28 498 (10.4%) of all SOK donors tested positive for HCV. Non-SOK donors were refused by a median (interquartile range [IQR]) of 5 centers (3–10 centers) on behalf of a median (IQR) of 16 candidates (6–94 candidates), whereas SOK donors were refused by a median (IQR) of 9 centers (4–23 centers) on behalf of a median (IQR) of 122 candidates (16–1014 candidates). Among the SOKs, IRDs and donors who had HCV were refused by a median (IQR) of 7 (3–14) and 7 (3–16) centers, respectively; whereas donors with CIT >36 h or age >65 were refused by a median (IQR) of 40 (11–82) and 28 (10–62) centers, respectively (Table 3).

**TABLE 4.** Characteristics of centers analyzed for aggressiveness in offer acceptance rates

Transplant characteristics of centers included in aggressiveness analysis	All centers	Stratified by adjusted KDPI >85% donor acceptance quartile among offered centers				
		All offered KDPI >85%	Q1	Q2	Q3	Q4
n	193	192	48	48	48	48
Transplant volume during study period, median (IQR)	269 (148–546)	269 (149–547)	143 (83–206)	222 (98–355)	427 (249–587)	580 (304–779)
Waitlist volume during study period, median (IQR)	496 (274–865)	496 (277–867)	248 (173–507)	401 (256–682)	600 (391–1098)	718 (558–1175)
Non-SOK donors offered						
Offered, median (IQR)	482 (327–692)	483 (331–697)	442 (331–659)	424 (300–622)	550 (350–792)	535 (335–718)
Accepted, median (IQR)	59 (35–96)	60 (36–97)	38 (25–59)	50 (31–76)	72 (42–105)	97 (68–140)
Percent accepted, median (IQR)	12.5% (8.4%–19.2%)	12.5% (8.5%–19.4%)	8.6% (4.8%–14.1%)	11.8% (8.8%–16.5%)	23.8% (9.2%–19.9%)	18.2% (11.6%–24.4%)
SOK donors offered						
Offered, median (IQR)	2732 (1854–3739)	2736 (1860–3745)	2583 (1619–3250)	2485 (1469–3152)	2906 (2333–4079)	2978 (1942–4012)
Accepted, median (IQR)	179 (93–312)	181 (93–314)	89 (48–142)	154 (70–228)	254 (168–336)	379 (201–488)
Percent accepted, median (IQR)	7.2% (4.6%–10.8%)	7.2% (4.6%–10.8%)	3.7% (2.4%–6.5%)	6.1% (4.2%–8.6%)	7.8% (5.9%–11.2%)	11.6% (8.7%–14.7%)
KDPI >85% donors offered						
Offered, median (IQR)	423 (227–642)	423 (227–642)	378 (166–594)	302 (173–513)	500 (348–680)	459 (318–681)
Accepted, median (IQR)	9 (3–23)	9 (3–23)	1 (0–4)	5 (3–9)	18 (13–24)	32 (22–48)
Percent accepted, median (IQR)	2.6% (0.9%–4.9%)	2.6% (0.9%–4.9%)	0.3% (0%–0.7%)	1.7% (1.3%–2.2%)	3.5% (3%–4.2%)	7% (5.4%–8.3%)

IQR, interquartile range; KDPI, kidney donor profile index; SOK, suboptimal kidneys.

### Characteristics of Centers in Offer Analysis

During the study period (2 y and 9 mo), the median (IQR) transplant volume of these 193 centers was 269 (148–536), and median (IQR) kidney waitlist registration volume was 496 (274–865). The median (IQR) numbers of SOK donors offered to and accepted by centers were 2732 (1854–3739) and 179 (93–312), respectively, during the study period, with the median (IQR) percent accepted at 7.2% (4.6%–10.8%; Table 4).

### Center-level Variation in SOK Offer Acceptance

**FIGURE 3.**
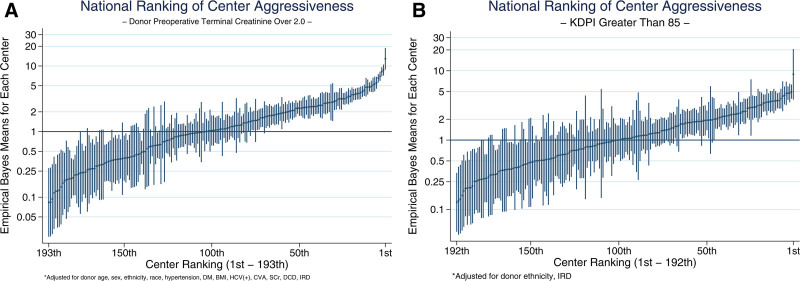
Illustration of center-specific odds in accepting suboptimal kidneys with creatinine >2.0 mg/dL or KDPI >85% vs the national average. A, Donor terminal SCr >2.0. B, KDPI. BMI, body mass index; CVA, cerebrovascular events or stroke; DCD, donated after circulatory death; DM, diabetes mellitus; HCV, hepatitis C virus; IRDs, increased risk donors; KDPI, kidney donor profile index; SCr, serum creatinine.

**FIGURE 4.**
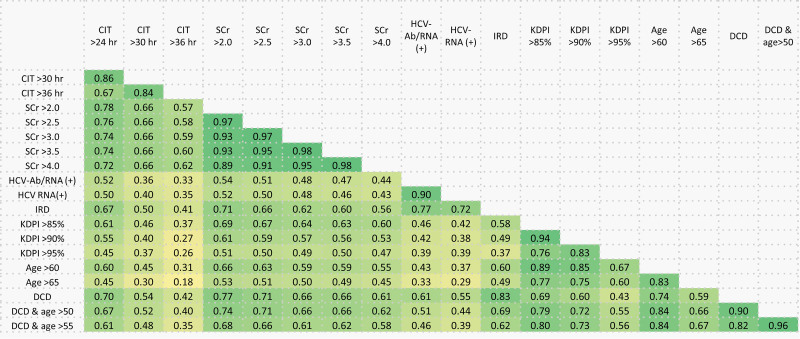
Correlations in center-level adjusted odds ratios of accepting various subtypes of suboptimal kidneys. CIT, cold ischemia time (h); DCD, donated after circulatory death; HCV-Ab, hepatitis C virus antibody; IRDs, increased risk donors; KDPI, kidney donor profile index; SCr, serum creatinine (mg/dL).

The mOR of all SOK donor categories with various cutoffs ranged from the lowest being IRD donor (mOR = 2.06), to the highest being CIT >36 h (mOR = 4.86; Table 3). As an example, for SOK donors whose terminal SCr was >2.0 mg/dL, a mOR of 2.86 means that on average, this SOK donor was 2.86-times more likely to be accepted by a randomly selected, more aggressive center compared with a less aggressive center. There was marked, center-level variation in the odds of accepting creatinine >2.0 mg/dL kidney offers (Figure 3A). mOR rose sharply from 2.52 for KDPI >85 offers to 3.58 for KDPI >85 offers with CIT >24 h, but the latter group represented only 3.5% of offers (Figure 3B). Correlations between how centers responded to different types of SOK donor offers points to patterns in center-level offer acceptance behavior (Figure 4). In some cases, there was strong correlation between centers’ willingness to accept different categories of SOK donors (eg, IRD and DCD; ρ = 0.83), for some there was moderate correlation (eg, IRD and CIT >24 h; ρ=0.67; Figure 4). Centers in the highest KDPI >85% kidney acceptance quartile had higher median transplant (580 versus 143) and waitlist volume (718 versus 248) than lowest-quartile centers (Table 4).

## DISCUSSION

In this national study of utilization and offer acceptance practices of SOK offers in the United States, we found substantial center-level variation in willingness to transplant SOKs in the current policy era. Center-level disproportionality in kidney utilization was greatest in transplants with prolonged CIT (>36 h), elevated donor SCr (>3.0 mg/dL), active donor HCV infection, or KDPI >95%. Our definition of “SOK” was broad: of all the DDKTs performed 2021–2023, 72.6% of donated kidneys came from donors with at least 1 characteristic that would classify them as being “suboptimal.” The utilization of SOKs was still more unequal than the non-SOKs. Some categories of SOK had relatively little variation in offer acceptance practices (eg, IRD, DCD), while others had much greater variation (eg, CIT >24 h, KDPI >85%, SCr >2.0 mg/dL, and DCD and age >60). Although there was correlation between centers’ willingness to accept different types of SOK, this correlation was only moderate for many types.

We found that the Gini coefficient of any SOK categories were higher than the Gini coefficient of the non-SOKs. This is consistent with the literature in that the utilization of SOK as DDKT was still more aggregated to certain centers than the utilization of non-SOKs.^5^ This was particularly true for SOKs fitting multiple categories, although donors meeting multiple SOK categories tended to represent a very small portion of offers. Some centers were more willing to accept DCD offers but less willing to accept offers with high CIT, while for other centers the reverse was true. Thus, labeling centers overall as “aggressive” or “nonaggressive” is an incomplete description of center decision-making; rather, each center has a unique, aggressiveness phenotype defined by varying degrees of willingness to transplant specific SOK subtypes. Different centers are aggressive in different ways.

In a prior article drawing on SRTR data from 2005 to 2009, our group reported inequality in the distribution of DDKTs performed using the Gini coefficient.^5^ Since that time, kidney allocation has changed substantially with the advent of KAS, and subsequently, KAS250.^15-18^ Additionally, research following the publication of our index study has demonstrated the survival benefit of transplants with various subtypes of SOK.^24,26-29,32,40,45,53,58-61^ This current work extends those findings by demonstrating that variability in center utilization of suboptimal organs has continued under KAS250. Moreover, the prior study was based solely on transplanted kidneys and did not measure variation in offer acceptance practices.^5^ Our current study has delved further into organ offer acceptance patterns, showing that variation in access to SOK is related to variation in decision-making. There are other factors that might contribute to the variation, such as center resources, center rurality, or weather conditions, which the centers had little or no control of. Consistent with existing literature using data from earlier periods,^9,10^ center-level variations persisted (although were relatively small) in accepting offers from older donors and IRD kidneys,^13,14^ whose definition changed since the prior analysis.^57^ Despite this, centers regardless of their acceptance patterns could all be providing appropriate care for their patients in the context of resources available. Centers in remote areas might lack the capacity to adequately support complex patients through prolonged delayed graft function and the increased risk of hospital readmission that entail after accepting SOK DDKTs.

Emerging evidence supporting utilization of SOKs has overwhelmingly demonstrated favorable posttransplant outcomes.^6,19,25,30,31,33-36,39,43,44,47-52,62-73^ Tomita et al^50^ showed that kidneys from DCD donors with stage 3 AKI could last for >10 y, and Orlando et al^49^ documented that kidneys from donors with AKI and CIT >40 h could last for >4 y posttransplant. Additionally, many studies have specifically demonstrated a survival benefit from accepting various types of SOK versus remaining on the waitlist in hopes of receiving a better offer.^23,24,26-29,32,40,43-46,53,57-61^ These kidneys could potentially benefit numerous waitlisted individuals. It is important for the centers to be intimately familiar with their own acceptance patterns,^74^ especially in comparison with other local and regional transplant centers, to evaluate continually if there are additional practice changes that might improve access and provide survival benefit for their waitlisted candidates. Program-Specific Reports released by the SRTR, along with the OPTN Membership & Professional Standards Committee’s adoption of a holistic set of performance metrics, including acceptance rates, are powerful first steps. Centers should be provided with institution-specific summarized information to assist in internal protocol development and offer decision-making.

Our findings presented here must be understood in the context of limitations of our study. In the offer analysis, CIT of both kidneys exceeding 24, 30, or 36 h was used as a proxy for a difficult-to-place kidney donor (likely because of the kidneys being suboptimal), not as a measure of the actual or projected CIT at time of offer, which was unavailable in data obtained for this study. Offer decisions for these kidneys were likely based on a combination of donor quality and CIT considerations, and the refusing centers may or may not have heavily factored CIT into their decisions. In turn, it stands to reason that a refusing center appearing earlier on the match run—for which the projected CIT at time of offer may very well have been <24 h (or 30–36 h)—would also have refused the same donor with excessive CIT. Thus, our inclusion of these refusals in the analysis is justified, despite not having the projected CIT at time of offer. Furthermore, our approach of requiring both kidneys to have CIT exceeding 24 h (or 30–36 h) is conservative with respect to defining kidney acceptance practices associated with excessive CIT, including fewer donors than if we included cases with 1 kidney’s CIT falling below the threshold. Therefore, we believe that our results reveal substantial and meaningfully defined center-level variation in acceptance of donors associated with high CIT at transplant. Inference from the offer analysis is broadly consistent with inference from the Gini coefficient, which relies on CIT of transplanted kidneys only, further bolstering our confidence in the results.

Our analysis was limited to SOK donors whose offer acceptances were within the same match run and were eventually accepted. Thus, the results could not be used to make inferences about the donors whose kidneys were accepted in multiple match runs, which could point to more nuanced center-level behaviors. However, our analyses included 99.3% of the SOK donors and should therefore accurately reflect the landscape of practices. Because a provisional acceptance does not necessarily indicate genuine interest in the organ, we did not treat these responses as either acceptances or refusals. In some cases, a provisional acceptance could indicate a center’s willingness to accept the donor for a suitable patient further down on the match run, if given the opportunity. Also, since offer data were summarized into 1 outcome (accepted or refused) for each center and donor, we did not account for recipient characteristics which might play a role in the center decision-making process (eg, sensitization, younger age).^27,28,75,76^ Our analysis did credit a center with acceptance of an SOK even if the offer was turned down for 1 or more patients before being accepted at that center. Nevertheless, our center-level offer acceptance rates do not account for centers’ ability to preemptively avoid receiving offers, for example by using the OPTN’s multifactorial offer filtering tool,^77^ since offers are not actually made but rather coded as bypasses. Finally, our study period included the COVID-19 pandemic. The COVID pandemic substantially altered center behavior in 2020,^78,79^ and COVID burden varied substantially by time and place.^80^ Thus, our findings might be less representative of center behavior prior to COVID-19. A nuanced evaluation of how the pandemic affected transplantation practices, however, is beyond the scope of this article. Nevertheless, COVID is not completely gone, and the pandemic might continue to impact offer acceptance behavior in the future, albeit likely to a lesser degree than during the first 2 y of the pandemic.

In summary, there is still substantial center-level variation in acceptances of SOK offers in the KAS250 era, and the degree of variation is much greater for some SOK subtypes compared with others. Centers should be cognizant of their SOK offer acceptance patterns and whether their rate of SOK offer acceptance is lower than the national average. This might help centers to accept more SOKs. This might improve access to DDKT for waitlisted patients at those centers and improve overall utilization of this valuable and scarce resource.

## Supplementary Material



## References

[1] Lentine KL Smith JM Hart A. OPTN/SRTR 2020 annual data report: kidney. Am J Transplant. 2022;22(Suppl 2):21–136. 35266618 10.1111/ajt.16982

[2] Husain SA King KL Pastan S. Association between declined offers of deceased donor kidney allograft and outcomes in kidney transplant candidates. JAMA Netw Open. 2019;2:e1910312. 31469394 10.1001/jamanetworkopen.2019.10312 PMC6724162

[3] Bowring MG Massie AB Craig-Schapiro R. Kidney offer acceptance at programs undergoing a systems improvement agreement. Am J Transplant. 2018;18:2182–2188. 29718565 10.1111/ajt.14907 PMC6117205

[4] Office of the Federal Register, National Archives and Records Administration. 84 Medicare and Medicaid Programs; Regulatory Provisions To Promote Program Efficiency, Transparency, and Burden Reduction; Fire Safety Requirements for Certain Dialysis Facilities; Hospital and Critical Access Hospital (CAH) Changes To Promote Innovation, Flexibility, and Improvement in Patient Care, 84 FR 51732, September 30, 2019, 51732–51834. https://www.federalregister.gov/documents/2019/09/30/2019-20736/medicare-and-medicaid-programs-regulatory-provisions-to-promote-program-efficiency-transparency-and. Accessed September 12, 2012.

[5] Garonzik-Wang JM James NT Weatherspoon KC. The aggressive phenotype: center-level patterns in the utilization of suboptimal kidneys. Am J Transplant. 2012;12:400–408. 21992578 10.1111/j.1600-6143.2011.03789.x

[6] Liu C Hall IE Mansour S. Association of deceased donor acute kidney injury with recipient graft survival. JAMA Netw Open. 2020;3:e1918634. 31913491 10.1001/jamanetworkopen.2019.18634 PMC6991314

[7] Zhou S Massie AB Holscher CM. Prospective validation of prediction model for kidney discard. Transplantation. 2019;103:764–771. 30015701 10.1097/TP.0000000000002362 PMC6330256

[8] Bowring MG Shaffer AA Massie AB. Center-level trends in utilization of HCV-exposed donors for HCV-uninfected kidney and liver transplant recipients in the United States. Am J Transplant. 2019;19:2329–2341. 30861279 10.1111/ajt.15355 PMC6658335

[9] Brennan C Husain SA King KL. A Donor utilization index to assess the utilization and discard of deceased donor kidneys perceived as high risk. Clin J Am Soc Nephrol. 2019;14:1634–1641. 31624140 10.2215/CJN.02770319 PMC6832051

[10] King KL Husain SA Schold JD. Major variation across local transplant centers in probability of kidney transplant for wait-listed patients. J Am Soc Nephrol. 2020;31:2900–2911. 33037131 10.1681/ASN.2020030335 PMC7790218

[11] Massie AB Stewart DE Dagher NN. Center-level patterns of indicated willingness to and actual acceptance of marginal kidneys. Am J Transplant. 2010;10:2472–2480. 20977638 10.1111/j.1600-6143.2010.03294.x

[12] Huml AM Albert JM Thornton JD. Outcomes of deceased donor kidney offers to patients at the top of the waiting list. Clin J Am Soc Nephrol. 2017;12:1311–1320. 28751577 10.2215/CJN.10130916 PMC5544513

[13] Roth EM Haque OJ Yuan Q. Heterogeneity in transplant center responses to the minimum acceptance criteria across UNOS regions. Clin Transplant. 2022;36:e14551. 34843130 10.1111/ctr.14551

[14] Holscher CM Bowring MG Haugen CE. National variation in increased infectious risk kidney offer acceptance. Transplantation. 2019;103:2157–2163. 31343577 10.1097/TP.0000000000002631 PMC6703966

[15] Jackson KR Zhou S Ruck J. Pediatric deceased donor kidney transplant outcomes under the kidney allocation system. Am J Transplant. 2019;19:3079–3086. 31062464 10.1111/ajt.15419 PMC6834871

[16] Massie AB Luo X Lonze BE. Early changes in kidney distribution under the new allocation system. J Am Soc Nephrol. 2016;27:2495–2501. 26677865 10.1681/ASN.2015080934 PMC4978057

[17] Shelton BA Sawinski D Ray C. Decreasing deceased donor transplant rates among children (</=6 years) under the new kidney allocation system. Am J Transplant. 2018;18:1690–1698. 29333639 10.1111/ajt.14663

[18] Stern J Alnazari N Tatapudi VS. Impact of the 2014 kidney allocation system changes on trends in A2/A2B into B kidney transplantation and organ procurement organization reporting of donor subtyping. Clin Transplant. 2021;35:e14393. 34165821 10.1111/ctr.14393

[19] Stewart DE Kucheryavaya AY Klassen DK. Changes in deceased donor kidney transplantation one year after KAS implementation. Am J Transplant. 2016;16:1834–1847. 26932731 10.1111/ajt.13770

[20] Zhou S Massie AB Luo X. Geographic disparity in kidney transplantation under KAS. Am J Transplant. 2018;18:1415–1423. 29232040 10.1111/ajt.14622 PMC5992006

[21] Schantz K Gordon EJ Lee U. Patient and clinician perceptions of informed consent and decision making about accepting KDPI > 85 kidneys. Transplant Direct. 2022;8:e1254. 34934806 10.1097/TXD.0000000000001254 PMC8683202

[22] Savoye E Tamarelle D Chalem Y. Survival benefits of kidney transplantation with expanded criteria deceased donors in patients aged 60 years and over. Transplantation. 2007;84:1618–1624. 18165773 10.1097/01.tp.0000295988.28127.dd

[23] Arcos E Pérez-Sáez MJ Comas J; Catalan Renal Registry*. Assessing the limits in kidney transplantation: use of extremely elderly donors and outcomes in elderly recipients. Transplantation. 2020;104:176–183. 30985579 10.1097/TP.0000000000002748

[24] Cohen JB Potluri V Porrett PM. Leveraging marginal structural modeling with Cox regression to assess the survival benefit of accepting vs declining kidney allograft offers. Am J Transplant. 2019;19:1999–2008. 30725536 10.1111/ajt.15290 PMC6591028

[25] Quast LS Grzella S Lengenfeld T. Outcome of kidney transplantation using organs from brain-dead donors older than 75 years. Transplant Proc. 2020;52:119–126. 31901319 10.1016/j.transproceed.2019.11.013

[26] Yu S Long JJ Yu Y. Survival benefit of accepting kidneys from older donation after cardiac death donors. Am J Transplant. 2021;21:1138–1146. 32659036 10.1111/ajt.16198 PMC8547550

[27] Massie AB Luo X Chow EK. Survival benefit of primary deceased donor transplantation with high-KDPI kidneys. Am J Transplant. 2014;14:2310–2316. 25139729 10.1111/ajt.12830

[28] Bae S Massie AB Thomas AG. Who can tolerate a marginal kidney? Predicting survival after deceased donor kidney transplant by donor-recipient combination. Am J Transplant. 2019;19:425–433. 29935051 10.1111/ajt.14978 PMC6309666

[29] Jay CL Washburn K Dean PG. Survival benefit in older patients associated with earlier transplant with high KDPI kidneys. Transplantation. 2017;101:867–872. 27495758 10.1097/TP.0000000000001405 PMC5292097

[30] Park WY Kim JH Ko EJ. Impact of kidney donor profile index scores on post-transplant clinical outcomes between elderly and young recipients, a multicenter cohort study. Sci Rep. 2020;10:7009. 32332846 10.1038/s41598-020-64055-8 PMC7181596

[31] Dube GK Brennan C Husain SA. Outcomes of kidney transplant from deceased donors with acute kidney injury and prolonged cold ischemia time—a retrospective cohort study. Transpl Int. 2019;32:646–657. 30712277 10.1111/tri.13406

[32] Lia D Singer P Nair V. DCD renal transplantation from donors with acute kidney injury. Transplantation. 2021;105:886–890. 32433240 10.1097/TP.0000000000003317

[33] Bauer J Grzella S Bialobrzecka M. Success of kidney transplantations from deceased donors with acute kidney injury. Ann Transplant. 2018;23:836–844. 30523243 10.12659/AOT.912660 PMC6298175

[34] Benck U Schnuelle P Kruger B. Excellent graft and patient survival after renal transplantation from donors after brain death with acute kidney injury: a case-control study. Int Urol Nephrol. 2015;47:2039–2046. 26498631 10.1007/s11255-015-1127-5

[35] Echterdiek F Kitterer D Dippon J. Outcome of kidney transplantations from >/=65-year-old deceased donors with acute kidney injury. Clin Transplant. 2022;36:e14612. 35148007 10.1111/ctr.14612

[36] Hall IE Akalin E Bromberg JS. Deceased-donor acute kidney injury is not associated with kidney allograft failure. Kidney Int. 2019;95:199–209. 30470437 10.1016/j.kint.2018.08.047 PMC6331055

[37] Hall IE Schroppel B Doshi MD. Associations of deceased donor kidney injury with kidney discard and function after transplantation. Am J Transplant. 2015;15:1623–1631. 25762442 10.1111/ajt.13144 PMC4563988

[38] Oikawa M Hatakeyama S Narita T. Safety and effectiveness of marginal donor in living kidney transplantation. Transplant Proc. 2016;48:701–705. 27234717 10.1016/j.transproceed.2015.09.067

[39] Si Nga H Takase HM Bravin AM. Good outcomes in kidney transplantation with deceased donor with acute kidney injury: donor’s age and not acute kidney injury predicts graft function. Transplant Proc. 2016;48:2262–2266. 27742275 10.1016/j.transproceed.2016.06.004

[40] Bowring MG Holscher CM Zhou S. Turn down for what? Patient outcomes associated with declining increased infectious risk kidneys. Am J Transplant. 2018;18:617–624. 29116674 10.1111/ajt.14577 PMC5863756

[41] Cannon RM Locke JE Orandi BJ. Impact of donor hepatitis C virus on kidney transplant outcomes for hepatitis C-positive recipients in the direct-acting antiviral era: time to revise the kidney donor risk index? Transplantation. 2020;104:1215–1228. 31517783 10.1097/TP.0000000000002949 PMC7083245

[42] Kasprzyk T Kwiatkowski A Wszola M. Long-term results of kidney transplantation from HCV-positive donors. Transplant Proc. 2007;39:2701–2703. 18021962 10.1016/j.transproceed.2007.09.021

[43] Scalea JR Barth RN Munivenkatappa R. Shorter waitlist times and improved graft survivals are observed in patients who accept hepatitis C virus+ renal allografts. Transplantation. 2015;99:1192–1196. 25340605 10.1097/TP.0000000000000479

[44] Shelton BA Sawinski D Mehta S. Kidney transplantation and waitlist mortality rates among candidates registered as willing to accept a hepatitis C infected kidney. Transpl Infect Dis. 2018;20:e12829. 29277956 10.1111/tid.12829

[45] Sibulesky L Leca N Limaye AP. Survival benefit in older patients transplanted with viremic hepatitis c positive kidneys when compared with high KDPI kidneys. Transplantation. 2022;106:2217–2223. 35675439 10.1097/TP.0000000000004179

[46] Cohen JB Eddinger KC Locke JE. Survival benefit of transplantation with a deceased diabetic donor kidney compared with remaining on the waitlist. Clin J Am Soc Nephrol. 2017;12:974–982. 28546439 10.2215/CJN.10280916 PMC5460711

[47] Hamano I Hatakeyama S Fujita T. Living kidney transplantation from marginal donors presents feasible donor renal function despite inferior recipient renal function. Transplant Proc. 2020;52:1723–1728. 32448670 10.1016/j.transproceed.2020.01.157

[48] Martins J Barreto S Bravo P. Kidney transplant from elderly donors: a center experience. Transplant Proc. 2020;52:1265–1268. 32217014 10.1016/j.transproceed.2019.12.051

[49] Orlando G Khan MA El-Hennawy H. Is prolonged cold ischemia a contraindication to using kidneys from acute kidney injury donors? Clin Transplant. 2018;32:e13185. 29285808 10.1111/ctr.13185

[50] Tomita Y Iwadoh K Ogawa Y. Single graft utilization from donors with severe acute kidney injury after circulatory death. Transplant Direct. 2018;4:e355. 10.1097/TXD.0000000000000768 PMC5908460 29707626

[51] Fletchinger T Jensen HK Wells A. Impact of prolonged cold ischemia time on one year kidney transplant outcomes. Transplant Proc. 2022;54:2170–2173. 36180253 10.1016/j.transproceed.2022.08.019

[52] Bikbov B Ruggenenti P Perna A. Long-term outcomes of kidney transplants from older/marginal donors: a cohort study. Nephron. 2021;145:642–652. 34130292 10.1159/000516534

[53] Garcia-Covarrubias A Morales J Espinosa V. Kidney donor profile index in a transplant center in Mexico. Transplant Proc. 2020;52:1136–1139. 32307147 10.1016/j.transproceed.2020.02.008

[54] Massie AB Kucirka LM Segev DL. Big data in organ transplantation: registries and administrative claims. Am J Transplant. 2014;14:1723–1730. 25040084 10.1111/ajt.12777 PMC4387865

[55] Hendele JB Limaye AP Sibulesky L. Misplaced emphasis, misunderstood risk: a cultural history of Public Health Service infectious disease guidelines. Curr Opin Organ Transplant. 2022;27:159–164. 35232929 10.1097/MOT.0000000000000954

[56] Naik AS Cibrik DM Sakhuja A. Temporal trends, center-level variation, and the impact of prevalent state obesity rates on acceptance of obese living kidney donors. Am J Transplant. 2018;18:642–649. 28949096 10.1111/ajt.14519

[57] Haugen CE Bowring MG Jackson KR. Offer acceptance patterns for liver donors aged 70 and older. Liver Transpl. 2022;28:571–580. 34559954 10.1002/lt.26309 PMC9627749

[58] Gill J Dong J Rose C. The risk of allograft failure and the survival benefit of kidney transplantation are complicated by delayed graft function. Kidney Int. 2016;89:1331–1336. 27165823 10.1016/j.kint.2016.01.028

[59] Bui K Kilambi V Mehrotra S. Functional status-based risk-benefit analyses of high-KDPI kidney transplant versus dialysis. Transpl Int. 2019;32:1297–1312. 31323698 10.1111/tri.13483 PMC6874710

[60] Arcos E Perez-Saez MJ Comas J; Catalan Renal Registry*. Assessing the limits in kidney transplantation: use of extremely elderly donors and outcomes in elderly recipients. Transplantation. 2020;104:176–183. 30985579 10.1097/TP.0000000000002748

[61] Pruett TL Vece GR Carrico RJ. US deceased kidney transplantation: estimated GFR, donor age and KDPI association with graft survival. EClinicalMedicine. 2021;37:100980. 34386752 10.1016/j.eclinm.2021.100980 PMC8343266

[62] Yemini R Rahamimov R Ghinea R. Long-term results of kidney transplantation in the elderly: comparison between different donor settings. J Clin Med. 2021;10:5308. 34830587 10.3390/jcm10225308 PMC8618615

[63] Helantera I Ibrahim HN Lempinen M. Donor age, cold ischemia time, and delayed graft function. Clin J Am Soc Nephrol. 2020;15:813–821. 32404337 10.2215/CJN.13711119 PMC7274280

[64] Heilman RL Smith ML Smith BH. Long-term outcomes following kidney transplantation from donors with acute kidney injury. Transplantation. 2019;103:e263–e272. 31205261 10.1097/TP.0000000000002792

[65] Kwon JA Park H Park SJ. Factors of acute kidney injury donors affecting outcomes of kidney transplantation from deceased donors. Transplant Proc. 2019;51:2575–2581. 31474451 10.1016/j.transproceed.2019.03.068

[66] Zheng YT Chen CB Yuan XP. Impact of acute kidney injury in donors on renal graft survival: a systematic review and meta-analysis. Ren Fail. 2018;40:649–656. 30396304 10.1080/0886022X.2018.1535982 PMC6225519

[67] Foroutan F Friesen EL Clark KE. Risk factors for 1-year graft loss after kidney transplantation: systematic review and meta-analysis. Clin J Am Soc Nephrol. 2019;14:1642–1650. 31540931 10.2215/CJN.05560519 PMC6832056

[68] Peters-Sengers H Houtzager JHE Idu MM. Impact of cold ischemia time on outcomes of deceased donor kidney transplantation: an analysis of a national registry. Transplant Direct. 2019;5:e448. 31165083 10.1097/TXD.0000000000000888 PMC6511440

[69] Jadlowiec CC Heilman RL Smith ML. Transplanting kidneys from donation after cardiac death donors with acute kidney injury. Am J Transplant. 2020;20:864–869. 31612611 10.1111/ajt.15653

[70] Fernandez HE Chiles MC Pereira M. Outcomes for potential kidney transplant recipients offered public health service increased risk kidneys: a single-center experience. Clin Transplant. 2018;32:e13427. 30329179 10.1111/ctr.13427

[71] Kayler L Yu X Cortes C. Impact of cold ischemia time in kidney transplants from donation after circulatory death donors. Transplant Direct. 2017;3:e177. 28706980 10.1097/TXD.0000000000000680 PMC5498018

[72] Forbes RC Feurer ID LaNeve D. Increasing kidney donor profile index sequence does not adversely affect medium-term health-related quality of life after kidney transplantation. Clin Transplant. 2018;32:e13212. 29377273 10.1111/ctr.13212 PMC5933873

[73] Ruck JM Segev DL. Expanding deceased donor kidney transplantation: medical risk, infectious risk, hepatitis C virus, and HIV. Curr Opin Nephrol Hypertens. 2018;27:445–453. 30169460 10.1097/MNH.0000000000000456 PMC6352990

[74] Mohan S Chiles MC. Achieving equity through reducing variability in accepting deceased donor kidney offers. Clin J Am Soc Nephrol. 2017;12:1212–1214. 28751578 10.2215/CJN.06220617 PMC5544510

[75] Schold JD Huml AM Poggio ED. A tool for decision-making in kidney transplant candidates with poor prognosis to receive deceased donor transplantation in the United States. Kidney Int. 2022;102:640–651. 35760150 10.1016/j.kint.2022.05.025

[76] Schold JD Meier-Kriesche HU. Which renal transplant candidates should accept marginal kidneys in exchange for a shorter waiting time on dialysis? Clin J Am Soc Nephrol. 2006;1:532–538. 17699256 10.2215/CJN.01130905

[77] King KL Husain SA Cohen DJ. The role of bypass filters in deceased donor kidney allocation in the United States. Am J Transplant. 2022;22:1593–1602. 35090080 10.1111/ajt.16967 PMC12989818

[78] Boyarsky BJ Po-Yu Chiang T Werbel WA. Early impact of COVID-19 on transplant center practices and policies in the United States. Am J Transplant. 2020;20:1809–1818. 32282982 10.1111/ajt.15915 PMC7262146

[79] Boyarsky BJ Ruck JM Chiang TP. Evolving impact of COVID-19 on transplant center practices and policies in the United States. Clin Transplant. 2020;34:e14086. 32918766 10.1111/ctr.14086

[80] Massie AB Werbel WA Avery RK. Quantifying excess deaths among solid organ transplant recipients in the COVID-19 era. Am J Transplant. 2022;22:2077–2082. 35294799 10.1111/ajt.17036 PMC9111343

