# Supplementary material for: Quantifying Center-level Aggressiveness in Transplanting Suboptimal, Deceased Donor Kidneys in the United States
Source: Transplant Direct. 2026 Jan 12;12(2):e1886. doi: 10.1097/TXD.0000000000001886 (PMC12795041; doi:10.1097/TXD.0000000000001886)
Supplement: Supplementary file 1 [file txd-12-e1886-s001.pdf]

Table S1: The Characteristics of Sub-Optimal Kidney Donors Included in the Offer Analyses used to Estimate Center-Level Median Odds Ratios

Donor Characteristics	non-SOK Donors	SOK Donors
n	9,031	19,467
Age, median years (IQR)	38 (26, 48)	42 (32, 54)
Female, n (%)	3,571 (39.5%)	6,700 (34.4%)
Race, n (%)		
Asian	275 (3.0%)	412 (2.1%)
Black	1,513 (16.8%)	2,538 (13.0%)
Multiracial, Native American, Pacific	169 (1.9%)	313 (1.6%)
White	7,068 (78.3%)	16,196 (83.2%)
Unreported	6 (0.1%)	8 (<0.1%)
Hispanic, n (%)	1,747 (19.3%)	2,576 (13.2%)
BMI, median (IQR)	27.2 (23.3, 31.8) (n=8,888)	27.7 (23.9, 32.5) (n=19,053)
CIT, median (IQR)	15.7 (12.0, 19.2)	18.9 (14.9, 23.1) (n=19,436)
CIT > 24-hrs, n (%)	n/a	4,262 (21.9%)
CIT > 30-hrs, n (%)	n/a	1,259 (6.5%)
KDPI, median (IQR)	31 (14, 52)	51 (30, 73) (n=19,466)
HTN, n (%)	4,583 (50.8%)	9,890 (51.7%)
DM, n (%)	4,677 (51.8%)	7,826 (40.6%)
SCr, median (IQR)	0.9 (0.7, 1.2)	1.0 (0.7, 1.8)
Died due to CVA, n (%)	2,142 (23.7%)	3,702 (19.0%)
HCV, n (%)	n/a	2,955 (15.2%)
IRD by CDC, n (%)	n/a	5,499 (28.5%)

abbreviations: SOK: suboptimal kidneys; IQR: inter-quartile range; BMI: body mass index; CIT: cold ischemia time; KDPI: kidney-donor profile index; HTN: hypertension; DM: diabetes mellitus; SCr: terminal serum creatinine; CVA: cerebrovascular events or stroke; IRD: increased-risk donors; CDC: Center of Disease Control
